# Which demographic characteristics are associated with willingness to take part in recontact studies? A cross-sectional study

**DOI:** 10.1371/journal.pone.0335986

**Published:** 2025-11-04

**Authors:** Zara Kayani, Andrew Willis, Safoora Gharibzadeh, Kamlesh Khunti, Ash Routen

**Affiliations:** 1 Diabetes Research Centre, Leicester General Hospital, University of Leicester, Leicester, United Kingdom; 2 HRB Clinical Research Facility & School of Public Health, University College Cork, Ireland; 3 Leicester Real World Evidence Unit, Diabetes Research Centre, Leicester General Hospital, University of Leicester, Leicester, United Kingdom; National Institutes of Health, University of the Philippines Manila / De La Salle University, PHILIPPINES

## Abstract

**Background:**

Lower rates of participation in research by ethnic minority groups and socioeconomically deprived populations has led to efforts to develop recruitment strategies which aim to address this imbalance. Little is known about participation in recontact studies (where existing research participants are recruited to further studies). Identifying factors which predict rates of participation and retention is crucial to ensure the benefits of diversified recruitment are realised upon study completion.

**Methods:**

This secondary data analysis utilised data from the Multi-Ethnic Lifestyle Study (MELS) which was a multi-centre study. Modified Poisson regression models were applied in Stata version 18.0 to examine differences in demographic characteristics (age, gender, ethnicity, migration status, education, employment) between existing MELS participants who did and did not consent to be recontacted for future health research, (2) consent to link study data to their health records, (3) and consent to share research data with universities and the NHS.

**Results:**

A total of 6147 participants (mean age: 51.9 years, ± 17.12) were included in the analysis. Older age, higher education level (A levels or above compared to GCSEs) participants were more likely to consent to be recontacted, and to agree to link study data to their health records. South Asian participants were less likely to consent to recontact (PR = 0.79, 95% CI [0.71–0.88], p < 0.001) and data linkage (0.72, 95% CI [0.64–0.82], p < 0.001) compared to White participants. As the number of long-term conditions increased, so did the likelihood of consenting to re-contact (PR = 1.14, 95% CI [1.04–1.23], p = 0.002) and data linkage (PR = 1.13, 95% CI [1.04–1.22], p = 0.003). For data linkage, participants who were not born in the UK, compared to UK born participants, were less likely to consent to having their data linked to health records, although the difference was not statistically significant (PR = 1.06, 95% CI [0.94–1.20], p = 0.316).

**Conclusions:**

Our findings highlight that willingness to participate in health research studies, consent to data linkage, and data sharing varies across demographic groups. Inclusive recruitment and retention strategies must be developed to encourage participation and retention in follow-up studies, especially among historically underserved groups.

## Introduction

Health research, and clinical trials which aim to influence and inform treatment decisions should recruit populations that match, as closely as possible, the populations who would be eligible for these same treatments if offered in routine care [1,2]. This should explicitly include groups considered ‘underserved’ due to underrepresentation in research, but who often experience a higher disease burden [2,3]. Despite recent efforts to improve recruitment and retention rates for research overall, groups traditionally underserved by health research, such as ethnic minority populations, low socioeconomic status groups and individuals with disabilities are still underrepresented in studies impacting external validity and increasing research waste [4–6]. The reasons driving underrepresentation of underserved groups in health research overall, include language barriers [7], financial issues [8], medical mistrust [9], experiences of racism and discrimination [10] and lack of awareness [11].

Previous research has reported that sociodemographic characteristics such as, age, gender, and ethnicity are predictors of whether an individual participates in a health research study [12,13]. A meta-analysis of 29 UK-based COVID-19 trials reported that 84.8% of participants were White, with Asian, Black and Mixed ethnic groups underrepresented, despite ethnic minority groups having an increased risk of severe disease and premature mortality from SARS-CoV-2 [14]. Additionally, all 30 UK-based COVID-19 vaccine trials *(n = 118,912)* underrepresented ethnic minority groups when compared to national population ethnicity data; differences were most marked in Black (1% 95% CI [0.6–1.5%] vs 3.3%) and Asian (5.8% 95% CI[4.4–7.6%] vs 7.5%) groups [14]. Despite the increased prevalence and severity of COVID-19, vaccine uptake in ethnic minority groups was also found to be lower than White ethnicities in the UK [15,16].

Aside from ethnicity, other sociodemographic factors may influence participants willingness to participate in health research. A study in a Danish population of employees (*n = 50,806*) found that women were more likely to participate than men, and participation increased with higher annual income and educational level [17]. Individuals with higher education levels may have higher health literacy, a greater understanding of what research entails, and a greater interest in research overall [18]. Lower educational levels are associated with difficulty in understanding the research process, including the informed consent processes [19,20].

Other factors such as having multiple long-term health conditions can also affect individuals’ willingness to participate in research. In an online survey study of 2150 participants based in the US, a greater proportion of individuals with two chronic diseases were more willing to participate in clinical research studies (53%) compared to those individuals with no chronic diseases (41%) [21]. This could be due to increased engagement with healthcare services, having a greater interest in improving their own health, and wanting to participate in research that could impact their quality of life [22].

Aside from the initial challenges in recruiting participants, there are also challenges in retaining participants. Poor retention rates of participants can potentially bias outcomes, negatively impacting the generalisability and validity of a study [23]. In the UK, trial methodologists identified clinical trial retention as one of the top three priority areas [24]. A review consisting of 6 European large, multicentre randomised controlled trials (*n = 7612*) concluded that barriers to retaining older participants in clinical trials included, high rates of comorbidities with high symptom burden, difficulties in accessing facilities, number of study visits and long durations for trial participation [25].

A meta-ethnographic synthesis found that participant’s decisions to discontinue trial participation included, being ‘too well’ to continue engagement with the trial, non-acceptance of a diagnosis amongst those who were newly diagnosed, or being too ill to engage fully with research processes [26].

While there is an existing body of evidence detailing factors associated with willingness to participate in research, less is known on willingness to consent to recontact studies (where existing research participants already consenting to a study are recruited to further data collection or future studies). Understanding the association between sociodemographic characteristics and willingness to re-participate in health research studies is essential for developing and sustaining of targeted, inclusive recruitment strategies.

Therefore, this study aims to address the following research question: What are the associations between sociodemographic characteristics (age, gender, ethnicity, migration status, education, employment) and number of health conditions, and participants willingness to consent to recontact studies. Furthermore, a secondary aim is to determine how sociodemographic characteristics and number of health conditions may influence participants’ consent for NHS data linkage and data sharing.

## Methods

### Study design

We conducted an analysis of the Multi-Ethnic Lifestyle Study (MELS). The main aim of MELS is to investigate lifestyle behaviours and the development of chronic disease in a UK based population (S1 File). The MELS study aims to recruit 10,000 participants in total from 2019 to 2026, with the recruitment target set to increase.

Participants who were recruited to the MELS study between January 2019 and October 2023 were included in this analysis. Participants were recruited from a variety of settings, including GP practices, community centres and hospital clinics around the East Midlands from January 2019. Participants were required to be aged 18 years or over, willing to complete the MELS questionnaire (S2 File), either online or on paper and able to read and understand English. Convenience sampling, a non-probability sampling method, was used to gather the data. The MELS questionnaire was split into 6 sections including, patients demographics (age, gender, ethnicity, education, employment, home postcode, GP surgery) and health status, diet, sleep, physical activity level, lived environment and COVID-19.

This study will analyse demographic variables (age, gender, ethnicity), socioeconomic status (education, employment), and the number of multiple long-term conditions, all of which were collected through the questionnaire. Socioeconomic data was gathered using education, employment and postcode as measures. As part of the informed consent process, the questionnaire included additional yes/no statements where participants could consent to be contacted in the future about further research, consent to link research data to NHS health records and consent to anonymous data sharing with universities, the NHS and other research organisations both in the UK and abroad.

### Statistical analysis

Data were analysed using Stata version 18.0. The data were cleaned, and the categories of ethnicity, education, and employment were combined to increase the number of cases available for analysis. After consultation with colleagues at the Office for National Statistics (ONS) in the UK, ethnicity was reclassified from 18 categories to 3 categories (White, South Asian and Other). Education was classified from 7 categories to 6 categories (None, GCSE or equivalent, A levels or equivalent, undergraduate degree, postgraduate degree, and other). Additionally, employment was reduced from 9 categories to 3 categories including, employed, unemployed, and retired (S3 File). There was insufficient postcode data to allow for calculation and analysis of Index of Multiple Deprivation, hence this variable was not included in the study. A new variable (number of multiple-long term conditions) was created which consisted of the total number of health conditions that participants had indicated on the questionnaire from 0 to 6 or more. Descriptive statistics were generated and categorical variables were reported in a table format using counts and percentages. Continuous variables were reported using mean and standard deviation.

Univariate and multiple modified Poisson regression analyses were undertaken to examine the relationship between the independent variables (age, gender, education, employment, ethnicity, migration status, number of MLTCs) and the outcome variables (willingness to be recontacted to participate in future research, data linkage and data sharing). Separate regression models were used for the three outcomes. Purposeful variable selection was followed to determine which of the independent variables should be included in the final Poisson regression models. A univariate analysis was conducted for each independent variable to determine its statistical significance. Variables which were statistically significant (p < 0.25) were included in the full Poisson regression model. Those variables not significant at the iterative stage were re-added to the multiple regression model, testing for significance of p < 0.10. Prevalence ratios (PRs) and their 95% confidence intervals (CIs) were obtained by exponentiating the regression coefficients from the modified Poisson models with robust error variance. The final analyses were conducted with 95% confidence intervals (CI) and p < 0.05 was considered statistically significant [27].

## Results

A total of 6147 participants were included in the analysis (mean age 51.9 years, ± 17.12). Of these participants, 62.3% were female and 60.8% of the sample identified as White. 28.1% of participants reported a diagnosis of two or more long term health conditions. Table 1 provides an overview of participant characteristics included in the analysis.

**Table 1:** Sociodemographic characteristics of participants included in the analysis.

Participant Characteristics	n (%)
**Age (years)**	
Mean age 51.9 years (±17.12)	
18–34	1072 (17.4)
35–64	2569 (41.8)
65 or older	1264 (20.6)
Missing	1242 (20.2)
**Gender**	
Male	1,994 (37.7)
Female	3,298 (62.3)
**Ethnicity**	
White	3,739 (60.8)
South Asian	1,160 (18.9)
Other	381 (6.2)
Missing	867 (14.1)
**Migration status (born in the UK?)**	
Yes	4122 (67.1)
No	1137 (18.6)
Missing	888 (14.3)
**Education**	
None	615 (10.0)
GCSE or equivalent	1,378 (32.4)
A Levels or equivalent	1,022 (49.1)
Undergraduate degree	1,138 (67.6)
Postgraduate degree	838 (81.2)
Other	191 (84.3)
**Employment**	
Employed	3,242 (52.7)
Unemployed	718 (11.7)
Retired	1,306 (21.3)
**Number of MLTCs**	
0 LTC	3,025 (49.1)
1 LTC	1,388 (22.6)
2 LTCs	897 (14.6)
3 LTCs	416 (6.8)
4 LTCs	200 (3.3)
5 LTCs	115 (1.9)
6 or more LTCs	106 (1.7)

### Re-contact and NHS data linkage

Overall, 54% of participants (*n = 3291*) consented to be re-contacted regarding opportunities to participate in further health research. Furthermore, 56% (*n = 3454*) of participants consented to having their data linked to their medical records. Results showed that older age was associated with a higher likelihood of consenting to be re-contacted for future research studies (PR = 1.08, 95% CI [1.05–1.11], p < 0.001) and data linkage to NHS records (PR = 1.07, 95% CI [1.04–1.10], p < 0.001). Participants with higher education levels (A levels or above compared to GCSEs) showed a 10% increased likelihood of consenting to re-contact and data linkage (PR = 1.10, 95% CI [0.98–1.22], p = 0.12; see Table 2 for re-contact and Table 3 for data linkage).

**Table 2:** Participant’s willingness to be recontacted about participating in future health research: results of the modified Poisson regression analyses.

	*PR (95%CI)*	*p- value*
**Age**	1.078 (1.05–1.11)	p < 0.001
**Gender**		
Male (ref)		
Female	1.072 (0.99–1.16)	p = 0.091
**Ethnicity**		
White (ref)		
South Asian	0.793 (0.71–0.88)	p < 0.001
Other	0.891 (0.76 - 1.05)	p = 0.158
**Education**		
None	0.921 (0.80–1.06)	P = 0.237
GCSE or equivalent (ref)		
A Levels or equivalent	1.096 (0.98–1.23)	P = 0.116
Undergraduate degree	1.152 (1.03–1.29)	P = 0.013
Postgraduate degree	1.135 (1.01–1.28)	P = 0.041
Other	1.119 (0.92–1.37)	P = 0.270
**MLTCS**		
2 or more MLTCs	1.138 (1.04–1.23)	0.002

**Table 3:** Participant’s willingness to consent to data linkage to medical records: results of the modified Poisson regression analyses.

	*PR (95% CI)*	*p- value*
**Age**	1.069 (1.04 - 1.10)	p < 0.001
**Gender**		
Male (ref)		
Female	1.009 (0.93–1.09)	p = 0.804
**Ethnicity**		
White (ref)		
South Asian	0.723 (0.64 - 0.82)	p < 0.001
Other	0.759 (0.64- 0.91)	p = 0.002
**Education**		
None	0.991 (0.87–1.13)	P = 0.894
GCSE or equivalent (ref)		
A Levels or equivalent	1.092 (0.98–1.22)	P = 0.124
Undergraduate degree	1.223 (1.10–1.36)	P < 0.001
Postgraduate degree	1.188 (1.05–1.34)	P = 0.005
Other	1.104 (0.90–1.35)	P = 0.330
**Employment**		
Employed (ref)		
Unemployed	1.057 (0.94–1.19)	0.361
Retired	0.983 (0.88–1.10)	0.779
**UK born?**		
Yes (ref)		
No	1.062 (0.94–1.20)	0.316
**MLTCS**		
2 or more MLTCs	1.129 (1.04–1.22)	0.003

The likelihood of both re-contact and data linkage was significantly lower among participants of South Asian ethnicity compared to those of White ethnicity (Re-contact: PR = 0.79, 95% CI [0.71–0.88], p < 0.001; Data linkage: PR = 0.72, 95% CI [0.64–0.82], p < 0.001). Additionally, individuals with multiple long-term conditions compared to those with one, were associated with higher likelihood of consenting to re-contact (PR = 1.14, 95% CI [1.04–1.23], p = 0.002) and data linkage (PR = 1.13, 95% CI [1.04–1.22], p = 0.003).

There was no significant association between whether participants were born in the UK or not and data linkage (PR = 1.06, 95% CI [0.94–1.20], p = 0.316; see Table 2 for re-contact and Table 3 for data linkage).

### Data sharing

74% of participants (*n = 4530*) consented to their data being shared with NHS, universities and other organisations.

There was no significant association found between data sharing to NHS, universities and other organisations and age (PR = 1.02, 95% CI [1.00–1.05], p = 0.100. The likelihood of willingness to consent to sharing data was lower in females than males (PR = 1.00, 95% CI [0.93–1.06], p = 0.96) and South Asian participants compared to white participants (PR = 0.85, 95% CI [0.77–0.94], p = 0.002). Participants with higher education levels (A levels or above compared to GCSEs) showed a 13% increased likelihood of consenting to data sharing (PR = 1.13, 95% CI [1.02–1.25], p = 0.015). An increased number of long-term conditions compared to just one was associated with higher likelihood of consenting to data sharing, albeit not statistically significant (PR = 1.01, 95% CI [0.99–1.04], p = 0.257). Individuals born outside of the UK were slightly less willing to consent to data sharing compared to their UK-born counterparts (PR = 0.93, 95% CI [0.84–1.03], p = 0.150; see Table 4 for data sharing).

**Table 4:** Participant’s willingness to consent to data sharing to universities. NHS and other organisations: results of the modified Poisson regression analyses.

	PR (95% CI)	p- value
**Age**	1.024 (1.00 - 1.05)	p = 0.100
**Gender**		
Male (ref)		
Female	0.998 (0.93–1.06)	p = 0.962
**Ethnicity**		
White (ref)		
South Asian	0.848 (0.77 - 0.94)	p = 0.002
Other	0.935 (0.81- 1.08)	p = 0.376
**Education**		
None	0.925 (0.82–1.05)	P = 0.219
GCSE or equivalent (ref)		
A Levels or equivalent	1.129 (1.02–1.25)	P = 0.015
Undergraduate degree	1.202 (1.09–1.32)	P < 0.001
Postgraduate degree	1.216 (1.10 - 1.35)	P < 0.001
Other	1.084 (0.91–1.30)	P = 0.380
**Employment**		
Employed (ref)		
Unemployed	0.973(0.87–1.08)	0.623
Retired	0.991 (0.89–1.10)	0.874
**Migration status (born in the UK?)**		
Yes (ref)		
No	0.926 (0.84–1.03)	0.150
**MLTCs**		
2 or more MLTCs	1.013 (0.99–1.04)	0.257

## Discussion

Our analysis showed that, of people taking part in a large cross-sectional questionnaire study, those from older age groups, those with a higher education level and those with more than one long-term health condition were more likely to give consent to be recontacted for future health research, and to grant access for linkage of their data to health records, and for their data to be shared with other universities and research organisations.

Previous research from the US reporting on willingness to be recontacted to participate in research found an association between self-identified White ethnicity and higher educational attainment with increased likelihood to consent [30]. Black/African American participants expressed a lower willingness to participate in research (56%) than White participants (70%). Furthermore, participants who were educated ‘up to some high school’ had lowest levels of willingness (51%) compared to those who had doctoral training (76%) [30]. These findings from a US population mirror results from this secondary data analysis. Participants with a higher level of education and higher level of health literacy had a higher level of interest in participating in health research [12].

The ATHENA COVID-19 study in Queensland, Australia aimed to recruit a cohort of patients with linked health information who were willing to be recontacted to participate in future research studies, including a long COVID study [28]. Among these 1155 patients whose contact details were available, 85% reached a consent decision. 73% of patient agreed to recontact and 69% agreed to data extraction, whereby their data would be extracted from their health practice and linked into other datasets for ethically approved studies. Altogether, 335 general practices took part and primary care data was successfully extracted with participants consent, highlighting the effectiveness of consent to recontact for the facilitation of recruitment to future studies [28].

Furthermore, participation in health research, including clinical trials often requires the ability to take on and withhold complex information relating to health conditions, new treatments, and the research process itself [7,29]. As a result, populations face greater barriers to participation, are often characterised by lower education levels [30,31], lower levels of written and spoken English [32,33], lower levels of health literacy [18] time constraints and caring responsibilities [34]. It is important to emphasise that researchers and institutions are responsible for addressing barriers relating to underrepresentation of underserved groups. Researchers must take active approaches to engage underserved groups through recommended strategies such as, tailored communication, translated materials and flexible data collection methods [29].

Regarding long-term health conditions, this study indicated that in all three regression models, individuals with a higher number of MLTCs were more willing to re-participate in research, have their data linked and data shared. This finding aligns with prior research focussing on rates of recruitment and retention [20]. It is important to note that our findings are directly applicable to individuals with MLTCs who have previously taken part in health research. Participants with an increased number of health conditions may be more willing to consistently participate in research due to perceiving participation as an important opportunity to contribute to advancements which improve their health outcomes, quality of life and access to new treatments [31].

Our analysis provides an insight into the characteristics of participants in health research and is beneficial for researchers to produce targeted interventions for specific groups. A major strength of using the MELS dataset was that it included a substantial proportion of those from ethnic minority groups (29.1%). In comparison to the UK census data (2021), approximately 25.5% of the UK population belongs to an ethnic minority group [33]. In relation to regional ethnic diversity, in the East Midlands, 20.5% of the population identified as belonging to an ethnic minority group [34]. The MELS dataset overrepresented ethnic minority groups, who are typically underserved in research studies. Also, this study examined a number of different variables (age, gender, socioeconomic status, multiple long-term conditions) in relation to participants willingness to consent to taking part in future studies, data linkage and data sharing.

Despite a large sample size, categories such as education and employment were collapsed to increase the statistical power, which may have obscured important differences in the analysis process. In the MELS dataset, there was missing data relating to participant’s age, ethnicity and migration status which may have impacted the validity of results. Moreover, there was limited data regarding participants postcodes so Index of Multiple Deprivation could not be included in the analysis. Given sufficient data, this would have provided further information on socioeconomic status overall, alongside education and employment and association with re-contact, data linkage and data sharing.

Despite efforts to ensure accessibility for underserved groups, it is certain that selection bias will have occurred in the recruitment the MELS study. Rather than regarding this as a weakness in the methods used in this analysis, our results will have more relevance to those planning and conducting recontact studies in the future, recruiting from existing cohorts, which will likely have similar selection bias. The population included in our analysis is one which has already given consent to participate in an observational questionnaire study, which matches the population to which our results will apply, giving a more accurate and reliable outcome estimate for consent and data linkage rates.The findings from our analysis provide valuable insights and may help inform the development of effective recruitment and retention strategies, specifically for the conduct of large-scale recontact studies associated with UK Biobank [35] and ‘Our Future Health,’ [36] which aim to engage diverse populations for the purpose of longitudinal research. Building on the results of our analysis, further work is necessary to explore specific factors such as trust, experiences with healthcare, perceived benefits, which may influence a participant’s decision to participate in multiple research studies. It is important to acknowledge that results from this study relate to health research participation more generally. We did not have data available to investigate the effects of study type on willingness to participate in future research. It is possible that rates of re-contact may differ according to study type and demand on participants (time, effort, inconvenience).

Having an understanding of these factors could provide valuable suggestions for researchers when designing health research studies, including clinical trials. This would contribute towards effective recruitment and retention of participants from a range of diverse groups in research. Moreover, exploring the barriers and enablers which influence a participant’s decision to re-participate and follow a trial through to the endpoint would be advantageous for the development of inclusive recruitment and retention strategies.

In conclusion, our analysis shows that differential rates in recruitment and retention in research are also evident in willingness to participate in further research, willingness for linkage of research data with medical records, and data sharing with universities and research organisations. Given the growing use of large biomedical databases for identification of eligible participants for clinical trials, there is a need to embed recruitment and retention strategies which address differential rates in participation, retention and willingness to consent to maximise the societal and health benefits of diverse research participation.

## Supporting information

S1 File MELS Summary. (DOCX)

S2 File Link to access the Multi-Ethnic Lifestyle Study questionnaire. (DOCX)

S3 File Original categories (from MELS questionnaire) and new categories created for statistical analysis. (DOCX)
