# Supplementary material for: Which demographic characteristics are associated with willingness to take part in recontact studies? A cross-sectional study
Source: PLoS One. 2025 Nov 4;20(11):e0335986. doi: 10.1371/journal.pone.0335986 (PMC12585038; doi:10.1371/journal.pone.0335986)
Supplement: S1 File — (DOCX) [file pone.0335986.s001.docx]

**S1 File – MELS summary**

| Multi-Ethnic Lifestyle Study (MELS) | Questionnaire (Online and paper based) |
| --- | --- |
| Aim | To investigate lifestyle behaviours and the development of chronic disease in an East Midlands based population. |
| Recruitment dates | January 2019 - ongoing |
| Recruitment | 6147 (as of October 2023) |
| Recruitment Target | 10,000 + |
