# Supplementary material for: Which demographic characteristics are associated with willingness to take part in recontact studies? A cross-sectional study
Source: PLoS One. 2025 Nov 4;20(11):e0335986. doi: 10.1371/journal.pone.0335986 (PMC12585038; doi:10.1371/journal.pone.0335986)
Supplement: S2 File — (DOCX) [file pone.0335986.s002.docx]

**S2 File – Link to access the Multi-Ethnic Lifestyle Study questionnaire**

[COVID19_Multi-Ethnic_Lifestyle_Study_BME_v3.pdf](https://static1.squarespace.com/static/5c2ccb867c93271f5edc9718/t/5f6b39158bfa6423aab9dd0f/1600862486893/COVID19_Multi-Ethnic_Lifestyle_Study_BME_v3.pdf)
