# Supplementary material for: Which demographic characteristics are associated with willingness to take part in recontact studies? A cross-sectional study
Source: PLoS One. 2025 Nov 4;20(11):e0335986. doi: 10.1371/journal.pone.0335986 (PMC12585038; doi:10.1371/journal.pone.0335986)
Supplement: S3 File — (DOCX) [file pone.0335986.s003.docx]

**S3 File - Original categories (from MELS questionnaire) and new categories created for statistical analysis**

| Original categories | New categories |
| --- | --- |
| ***Ethnicity***   1. White - Irish 2. White - Gypsy or Irish Traveller 3. White - European 4. Any other White background please describe 5. Asian/Asian British - Indian 6. Asian/Asian British - Pakistani 7. Asian/Asian British - Bangladeshi 8. Asian/Asian British - Chinese 9. Any other Asian/Asian British, (please describe) 10. Black/Black British - African 11. Black/Black British - Caribbean 12. Any other Black/African/Caribbean background, (please describe) 13. Mixed - Asian and White 14. Mixed - Black African and White 15. Mixed - Black Caribbean and White 16. Other Mixed background, (please describe) 17. Arab 18. Any other background | ***Ethnicity***   1. White 2. South Asian 3. Other |
| ***Education***   1. None 2. GCSE or equivalent 3. A levels or equivalent 4. Undergraduate degree 5. Postgraduate degree 6. Doctorate 7. Other | ***Education***   1. None 2. GCSE or equivalent 3. A levels or equivalent 4. Undergraduate degree 5. Postgraduate degree 6. Other |
| ***Employment***  1. Employed full time  2. Employed part time  3. Self-employed  4. Unemployed  5. Student  6. Seeking work  7. Retired  8. Long term sick  9. Homemaker  10. Other | ***Employment***   1. Employed 2. Unemployed 3. Retired |
